# Comparative Transcriptome Analysis Reveals Effects of Exogenous Hematin on Anthocyanin Biosynthesis during Strawberry Fruit Ripening

**DOI:** 10.1155/2016/6762731

**Published:** 2016-12-15

**Authors:** Yi Li, Huayin Li, Fengde Wang, Jingjuan Li, Yihui Zhang, Liangju Wang, Jianwei Gao

**Affiliations:** ^1^Institute of Vegetables and Flowers, Shandong Academy of Agricultural Sciences and Shandong Key Laboratory of Greenhouse Vegetable Biology and Shandong Branch of National Vegetable Improvement Center, Jinan 250100, China; ^2^College of Horticulture, Nanjing Agricultural University, Nanjing 210095, China

## Abstract

Anthocyanin in strawberries has a positive effect on fruit coloration. In this study, the role of exogenous hematin on anthocyanin biosynthesis was investigated. Our result showed that the white stage of strawberries treated with exogenous hematin had higher anthocyanin content, compared to the control group. Among all treatments, 5 *μ*M of hematin was the optimal condition to promote color development. In order to explore the molecular mechanism of fruit coloring regulated by hematin, transcriptomes in the hematin- and non-hematin-treated fruit were analyzed. A large number of differentially expressed genes (DEGs) were identified in regulating anthocyanin synthesis, including the DEGs involved in anthocyanin biosynthesis, hormone signaling transduction, phytochrome signaling, starch and sucrose degradation, and transcriptional pathways. These regulatory networks may play an important role in regulating the color process of strawberries treated with hematin. In summary, exogenous hematin could promote fruit coloring by increasing anthocyanin content in the white stage of strawberries. Furthermore, transcriptome analysis suggests that hematin-promoted fruit coloring occurs through multiple related metabolic pathways, which provides valuable information for regulating fruit color via anthocyanin biosynthesis in strawberries.

## 1. Introduction

Strawberry (*Fragaria × ananassa* Duch.) is one of the most popular fruits with global economic importance [1]. Because of its appealing red coloration and abundant nutrition, strawberries are highly sought after by consumers [2, 3]. These qualities are partially due to the high anthocyanin content in strawberry. Anthocyanins have a high antioxidant activity [4]. Research suggests that anthocyanins have potential health benefits for a variety of conditions including cardiovascular disorders, advanced age-induced oxidative stress, inflammatory response [5], and diverse degenerative diseases [6, 7]. Increasing anthocyanin content in strawberries has been a relevant research topic in recent years.

Biosynthesis of anthocyanins is a complex biological process which is affected by genetic, developmental, and environmental factors [8]. Over the past few years, most structural genes encoding enzymes in the anthocyanin biosynthetic pathways have been isolated and characterized in strawberries. The first group of structural genes involved in these pathways includes phenylalanine ammonia lyase (PAL), chalcone synthase (CHS), flavanone 3-hydroxylase (F3H), dihydroflavonol-4-reductase (DFR), leucoanthocyanidin reductase (LAR)/anthocyanidin synthase (ANS), and UDP-glucose flavonoid 3-O-glucosyltransferase (UFGT). These structural genes comprise the pathways needed for the synthesis of anthocyanins [9]. These genes are regulated primarily by the Myb/bHLH/WD40 (MBW) complex in many plants [10, 11].

It has been shown that anthocyanin synthesis increases rapidly after the white stage in strawberries [12]. Phytohormones such as abscisic acid (ABA), cytokinin (CTK), and ethylene and methyl jasmonate (JA) also play an important role in regulating the color development process in strawberries by increasing anthocyanin accumulation [12, 13]. Auxin and gibberellins (GAs) are known to reduce anthocyanin biosynthesis during the color development in fruit [8].

Light is one of the most important environmental factors regulating anthocyanin biosynthesis [14]. Phytochromes, which act as photoreceptors, play an important role in light stimulation during the development of strawberries [15]. Phytochromes are homodimeric chromoproteins where each holophytochrome is composed of a phytochrome protein covalently bound to a linear tetrapyrrole chromophore phytochromobilin (PΦB). PΦB acts as a light-receiving antenna for phytochrome. PΦB is synthesized in the plastid from heme catalyzed by heme oxygenase (HO) and subsequently phytochromobilin synthase [16]. Heme oxygenase 1 (HO1) is crucial to this process and acts as a rate-limiting enzyme in the biosynthesis of PΦB. HO1 catalyzes the oxygenation of heme to carbon monoxide, Fe^2+^, and biliverdin (BV) in plants [17]. Additionally, HO1 has been shown to play an important role in anthocyanin accumulation in plants. For example, tomato [18, 19] and* Arabidopsis* [20–22]* HO1* deficient mutants, which can not synthesize the phytochrome chromophore, have a major reduction in anthocyanin accumulation.

Hematin (C_34_H_33_O_5_N_4_Fe), a protoporphyrin complex, is an inducer and substrate of HO1 in animals and plants [17, 23]. Exogenous hematin was shown to alleviate mercury-induced oxidative damage in the roots of* Medicago sativa* [17], induce adventitious root numbers and root length of cucumbers [24], regulate* Brassica nigra* seed germination under nanosilver stress, and relieve etiolation in the leaves of wheat seedlings under complete darkness [25, 26]. These effects might be derived from a hematin induced HO1 enzymatic reaction product.

In this study, the effect of exogenous hematin on the white stage of strawberries was investigated by comparing the anthocyanin contents in the hematin-treated fruit and the control group. In order to gain insight into the underlying molecular mechanisms regulating fruit coloring in response to the hematin treatment, an analysis of mRNA expression profiles was performed using high-throughput sequencing. The results demonstrated that exogenous hematin could promote fruit coloring. Comparative transcriptome analyses may give us a better understanding of the mechanism of the coloring process in strawberry fruit.

## 2. Materials and Methods

### 2.1. Plant Materials, Growth Condition, and Hematin Treatments

**Figure 1:**
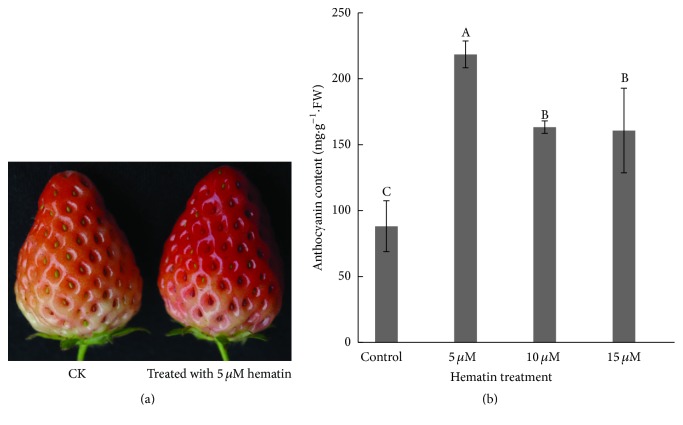
Fruit coloration and the anthocyanin content in the strawberry fruit. The color of the 5 *μ*M hematin-treated strawberry fruit was redder than the control (CK) at 48 h after treatment (a). The anthocyanin content in the strawberry fruit was measured after treatment with different concentrations of hematin (0, 5, 10, and 15 *μ*M) (b). Vertical bars represent standard errors; values with different letter are significantly different at *p* < 0.01.

Strawberry (*Fragaria × ananassa *Duch cv. Benihoppe), an octoploid (2*n* = 8*X* = 56) species, was planted under standard culture conditions in a greenhouse (30/15°C, 14/10 h day/night, relative humidity 50–80%). The maximum light intensity inside the greenhouse was 55,300 lux. The developmental stage of the strawberry fruit was divided into seven visual stages: small green (SG), big green (BG), degreening (DG), white (Wt), initial red (IR), partial red (PR), and full red (FR) [12]. Strawberry plants at the white stage (about 25 d after anthesis) were chosen to study the effect of hematin on fruit coloration in the study. To study the effect of different concentration of hematin on fruit coloration, white stage strawberry plants (*n* ≥ 50 for each treatment) were sprayed with 0, 1, 10, or 100 *μ*M hematin (H3281, Sigma-Aldrich, St. Louis, MO, USA), respectively [26]. Strawberry fruits (*n* ≥ 30 for each treatment) were harvested when the fruit entered the PR stage (48 h after treatment). The fruits were immediately frozen in liquid nitrogen and stored at −80°C for further analysis. We found the 10 *μ*M hematin-treated strawberries accumulated most anthocyanin among all treatments (Figure S1, in Supplementary Material available online at http://dx.doi.org/10.1155/2016/6762731). By using the same method to treat the fruits with 0, 5, 10, or 15-*μ*M hematin, we found the treatment by 5 *μ*M hematin was the optimal condition for increasing anthocyanin production in strawberry (Figure 1) (*p* < 0.01). The treatment with 5 *μ*M hematin was used for subsequent analysis. Three independent replicates were prepared for each treatment.

### 2.2. Determination of the Anthocyanin Content

The total anthocyanin content of the strawberries was determined using the method previously reported [6]. Absorbance was recorded on a Beckman DU640B spectrophotometer (Fullerton, CA, USA) at 510 and 700 nm for anthocyanin solutions in a pH 1.0 and pH 4.5 buffer, respectively. The calculated absorbance was obtained according to

(1) $$\begin{array}{c} A=\left[\;{\left(\;A_{510}-A_{700}\;\right)}_{\text{pH 1.0}}-{\left(\;A_{510}-A_{700}\;\right)}_{\text{pH 4.5}}\;\right]. \end{array}$$

The molar extinction coefficient is 26,900 as described in other studies (e.g., [6]). Anthocyanin concentrations were expressed in milligrams of cyanidin-3-galactoside equivalent per gram of fresh weight. Three independent replicates were conducted for each treatment.

### 2.3. BV Preparation and Assay

Strawberry fruit (1 g) was homogenized in a Potter-Elvehjem homogenizer using 1.2 mL ice-cold 0.25 M sucrose solution containing 1 mM phenylmethyl sulfonyl fluoride, 0.2 mM EDTA, and 50 mM potassium phosphate buffer (pH 7.4). Homogenates were centrifuged at 20,000 ×g for 20 min and chloroplasts were used for activity determination. BV was assayed as previously described [27]. The concentration of BV was estimated using a molar absorption coefficient at 650 nm of 6.25 mM^−1^ cm^−1^ in 0.1 M HEPES-NaOH buffer (pH 7.2). Three independent replicates were conducted for each treatment.

### 2.4. RNA Isolation and cDNA Library Construction

Total RNA isolation was carried out as previously described [28]. The procedure is briefly presented below. Strawberry fruits were ground into powder and mixed at a ratio of 0.5 g powder to 20 mL extraction buffer (200 mM Tris-HCl (pH 8.2), 100 mM LiCl, 50 mM ETA, 1.5% SDS, 2% PVP (Sigma, PCP40), 2% BSA (Sigma), and 10 mM DTT (Sigma)). A total of 200 *μ*L 10 mg/mL proteinase K (Merck, Darmstadt, Germany) was added to remove contaminating proteins. Total RNA was extracted using phenol/chloroform/isoamyl alcohol (25 : 24 : 1) and precipitated in a sodium acetate and ethanol mixture. The mixture was resuspended in an appropriate volume of DEPC-treated distilled water and then stored at −80°C for the next step. The quality and quantity of the total RNA were measured using a NanoDrop ND-1000 Spectrophotometer (NanoDrop, Wilmington, DE, USA). Only samples that met the criteria of 1.8 ≤ OD260/280 ≤ D2.0 and OD260/230 ≥ 1.8 and concentration ≥ 200 ng/*μ*L were used for sequencing. RNA samples of 30 strawberry fruits harvested in the same treatment group were pooled together for subsequent experiments.

RNA samples from two biological replicates were used for cDNA library construction and RNA-Seq at the Beijing Genomics Institute (BGI, Shenzhen, China). Total RNA samples were treated with DNase I (TaKaRa, Dalian, China) to remove any possible DNA contamination. The mRNA was enriched by using oligo (dT) magnetic beads (Illumina, San Diego, CA, USA) and cut into short fragments (about 200 bp). The first-strand cDNA was synthesized using a Superscript Preamplification System Kit (Gibco-BRL, Grand Island, NY, USA) as described in the manufacturer's instructions. The double stranded cDNA was purified with the oligo (dT) magnetic beads following the manufacturer's instructions. End repair was performed and adaptors were ligated to the ends of these fragments. Ligation products were purified using TAE-agarose gel electrophoresis. The fragments were then enriched by PCR amplification with an initial denaturing step at 98°C for 30 s, followed by 15 cycles of amplification (98°C for 10 s, 65°C for 30 s, and 72°C for 5 min) and a final extension at 72°C for 5 min. The PCR products were then purified using the oligo (dT) magnetic beads. DNA size, purity, and concentration were checked on an Agilent 2100 Bioanalyzer (Agilent, Santa Clara, CA, USA).

### 2.5. RNA Sequencing and Identification of Differentially Expressed Genes

RNA sequencing was performed using the ion proton platform at the Beijing Genomics Institute. The original sequence data were filtered to obtain clean reads for further analyses by removing short reads (less than 30 bp) and trimming adapters. Adapter reads were trimmed by first calculating the average quality of the first 15 bases from 3′-end until the average quality was larger than 10 and then removing the bases that were counted. The high-quality clean reads were mapped against the strawberry reference genome (http://strawberry-garden.kazusa.or.jp/) using Ion Torrent's mapping program (TMAP, version 0.2.3; https://github.com/iontorrent/TMAP). No more than two mismatches were allowed in the sequence alignment. Quality assessment of reads, statistics of alignment, sequencing saturation analysis, and randomness assessments were carried out subsequently to assess the quality of sequencing.

Gene expression levels were quantified by the software Sailfish [29]. Raw counts were normalized to Reads Per Kilobase of exon model per Million mapped reads (RPKM). Differential expression analysis was performed using the EBSeq package [30]. *Q* value < 0.05 and |log_2_⁡(fold  change)| > 1 were set as the threshold to identify significant differentially expressed genes. In statistics, correction for false positive errors was carried out using the FDR statistic [31].

### 2.6. Gene Ontology and Pathway Enrichment Analysis of Differentially Expressed Genes

Gene functions of strawberry were annotated according to the Gene Ontology (GO) standardized terms for molecular function, cellular component, and biological process using Blast2GO (https://www.blast2go.com/). Gene function was annotated using the following databases: NCBI nonredundant protein sequences, NCBI nonredundant nucleotide sequences, protein family, Clusters of Orthologous Groups of proteins, the manually annotated and reviewed protein sequence database, KEGG Ortholog database, and Gene Ontology. After GO annotation for DEGs, we performed GO functional classification for DEGs by the WEGO software [32] and analyzed the distribution of gene functions. We used the GO Term Finder tool (http://www.yeastgenome.org/help/analyze/go-term-finder) to search for significant shared GO terms.

(2) $$\begin{array}{c} p=1-\overset{m-1}{\underset{i=0}{\sum }}\frac{\left(\begin{array}{c} M\\ i \end{array}\right)\left(\begin{array}{c} N-M\\ n-i \end{array}\right)}{\left(\begin{array}{c} N\\ n \end{array}\right)}, \end{array}$$

where *N* is the number of all genes with GO annotation; *n* is the number of DEGs in *N*; *M* is the number of all genes that are annotated to certain GO terms; and *m* is the number of DEGs in *M*. The calculated *p* value was subjected to Bonferroni Correction [33]. The corrected *p* value < 0.05 was set as the threshold. GO terms fulfilling this condition are defined as significantly enriched GO terms in DEGs. The pathway enrichment analyses using the KEGG database (http://www.genome.jp/kegg/) were conducted subsequently to study the functions of differentially expressed genes identified between the control and the hematin-treated strawberry groups. The formula is the same as that in GO analysis. Here *N* is the number of all genes with KEGG annotation, *n* is the number of DEGs in *N*, *M* is the number of all genes annotated to specific pathways, and *m* is the number of DEGs in *M*.

### 2.7. Quantitative Real-Time PCR Analysis

Quantitative real-time PCR (qRT-PCR) experiments were conducted to assess the reliability of the RNA-Seq data [34]. Eleven pairs of gene specific primers were designed to verify the DEGs in the strawberry fruit (Table S1). Strawberry RNA was extracted from the fruit according to the previously detailed method. Total RNA was digested with DNase I for 30 min at 25°C to remove DNA contamination according to the manufacturer's instructions. The qRT-PCR was performed using a SYBR PCR master mix (TaKaRa, Dalian, China) on a Bio-Rad IQ-5 thermal cycler (Bio-Rad, Philadelphia, PA, USA). Three replicates of each sample were conducted to calculate the average Ct values. The relative expression level was calculated by the comparative 2^−ΔΔCt^ method [35, 36]. The significance was determined with the SPSS software (SPSS 17.0, IBM, Chicago, IL, USA) (*p* < 0.05).

## 3. Results and Discussion

### 3.1. Effects of Exogenous Hematin on Anthocyanin and Biliverdin Accumulation

**Figure 2:**
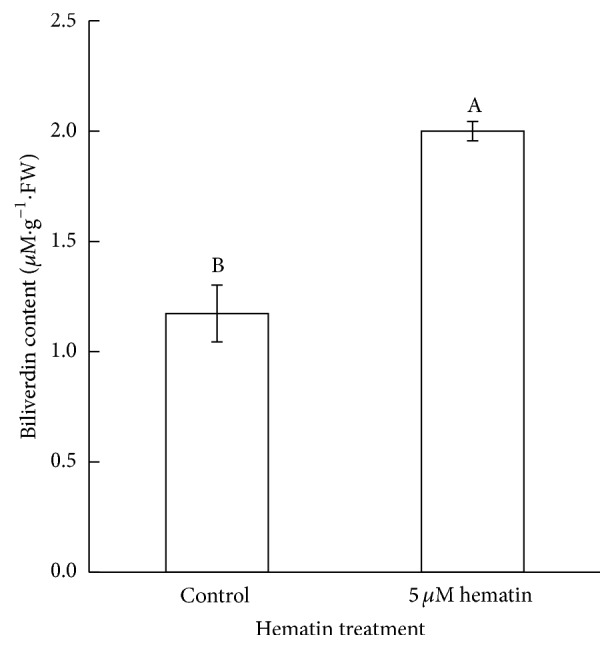
The BV content in strawberry fruit was measured after treatment with 0 and 5 *μ*M hematin. Vertical bars represent standard errors; values with different letter are significantly different at *p* < 0.01.

Hematin is an inducer and substrate of HO1 in animals and plants [17, 23]. In this study, strawberries at the Wt stage were treated with 0 *μ*M (control), 5, 10, or 15 *μ*M hematin. The results indicated that the anthocyanin content in the hematin-treated strawberries was more than the control after 48 h. The treatment by 5 *μ*M hematin was found to be the optimal condition for increasing anthocyanin production (Figure 1) (*p* < 0.01). The anthocyanin content in the 5 *μ*M hematin-treated strawberries was 2.5 times higher than that in the control. This is the first report that hematin could increase the anthocyanin production in fruit. We measured the expression of* FaHO-1* and the content of biliverdin (BV) which is the metabolite of heme oxygenase. We found that hematin could also significantly increase the expression of* FaHO-1* in strawberry fruit (Figure 4) and promote the accumulation of BV (Figure 2). These results indicate that hematin promotes anthocyanin accumulation through the heme metabolism pathway.

### 3.2. Sequences Assembly, Mapping, and Functional Annotation

High-throughput sequencing technology is utilized widely in analyzing gene expression in many organisms [37, 38]. In this study, approximately 10.8 Gbp raw tags were generated for each library. After eliminating adapters, ambiguous nucleotides, and low-quality sequences, a total of 34,618,832 and 45,464,123 clean reads between 150 and 200 nucleotides in length were obtained (Table 1). Over 85% of the clean tags from each library mapped to reference genes with less than 2 bp mismatches. Less than 29% of the clean tags from each library could not be aligned to any reference genes because of incomplete sequences, and these tags were designated as unknown. More than 67.3% of the clean tags in each library were mapped to a single gene, while less than 4.7% mapped to multiple reference genes. Unknown tags and tags mapped to multigenes were filtered out, and the unique clean tags that mapped to a single gene were retained for further DEG analysis.

In addition, the perfect match rates were 24.32%, 24.28%, 24.79%, and 24.35% in control 1, control 2, hematin 1, and hematin 2, respectively.

The saturation of the libraries with and without the hematin treatment was analyzed. The number of the detected genes became saturated at about 2 million reads (Figure S2). This indicates that the obtained reads are sufficient for complete transcriptome coverage. The randomness of RNA fragmentation in the four libraries was assessed for subsequent bioinformatics analysis. The results showed that the reads in each position of the reference gene were distributed evenly and demonstrated highly similar tendencies in all libraries (Figure S3).

### 3.3. Genes Differentially Regulated in Response to Exogenous Hematin

Based on sequencing results of the four mRNA libraries, approximately 29,000 genes (about 70% of the reference genes in the octoploid strawberry genome) were detected in each library. Additionally, correlations among genes based on RPKM between the two biological replicates were analyzed; the correlation coefficients (*R* ^2^) were high (0.93 and 0.95 for the control and hematin-treated group, resp.). Genes expressed in both replicates were screened. A total of 28,713, 28,999, 27,976, and 28,211 genes (Table 1) were expressed in the control and hematin-treated samples.

To reveal the molecular pathways regulating strawberry coloring in response to hematin, the DEGs between the control and hematin-treated fruit were analyzed. We compared the gene expression profiles between the control and the treated samples in both biological replicates. DEGs detected in both biological replicates were screened for subsequent analysis. A total of 1,080 (402 up- and 678 downregulated) genes were differentially expressed in the 5 *μ*M hematin-treated groups compared with the control groups. More genes were downregulated in the hematin-treated group compared to the control group.

**Figure 3:**
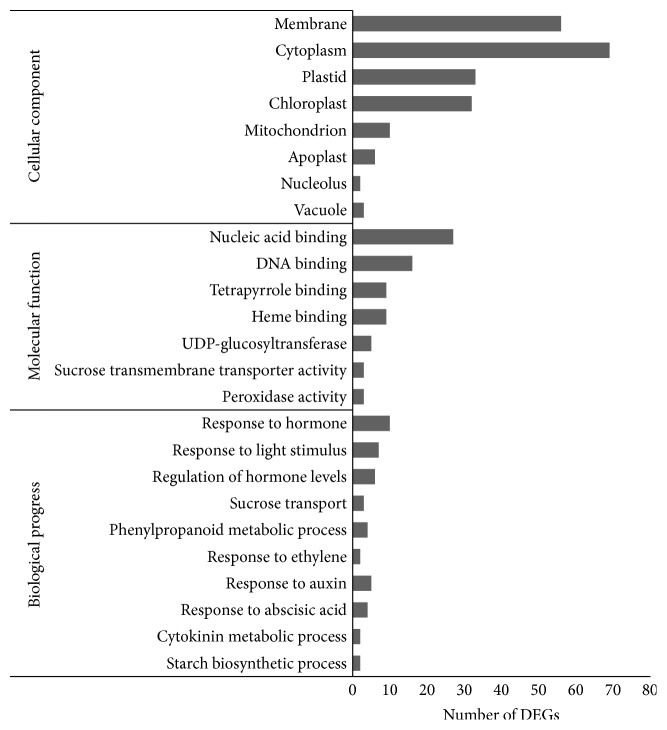
Gene classification based on Gene Ontology (GO) for DEGs in the strawberries treated by hematin.

To facilitate the global analysis of gene expression, a GO analysis was performed by mapping each differentially expressed gene into the records of the GO database. Gene Ontology (GO) enrichment analysis of the DEGs in the control and hematin-treated groups was performed to reveal the possible mechanisms under which hematin promotes anthocyanin biosynthesis. GO terms with *p* < 0.05 were represented among genes with significant changes in expression over a given interval. In this study, only three terms including anatomical structure arrangement, meristem structural organization, radial pattern formation were significantly enriched in biological process (Supplementary Table S2). Although most Go terms were not significantly enriched, they could provide a reference for further study. According to the GO cellular components, a large number of the DEGs were classified into main cell organelles, such as vacuole, nucleolus, mitochondrion, apoplast, chloroplast, plastid, cytoplasm, and membrane. In particular, many DEGs were assigned to the plastid and chloroplast (Figure 3), indicating that hematin has an important impact on the expression of plastid and chloroplast genes. It is probably because heme oxygenase 1 is a soluble plastid protein [22]. According to the GO molecular function, a large number of the DEGs were classified into UDP-glucosyltransferase, sucrose transmembrane transporter activity, nucleic acid binding, and DNA binding terms which are closely related to anthocyanin biosynthesis. Many DEGs were classified into heme binding, peroxidase activity, and tetrapyrrole binding (Figure 3). It is probably because hematin can increase the activity of HO1 and promote the degradation of hemoglobin [17]. In addition, anthocyanin accumulation in fruit is closely related to hormone [8] and carbohydrate biosynthesis [9]. According to the GO biological process, a large number of the DEGs were classified into regulation of hormone levels, cytokinin metabolic process, response to hormone, response to abscisic acid, response to auxin, and response to ethylene terms, indicating that the hormone-related DEGs are involved in regulating the anthocyanin biosynthesis in the hematin-treated fruit. Many DEGs were classified into starch and sucrose biosynthetic and transport process and phenylpropanoid metabolic process terms, indicating that hematin can promote anthocyanin accumulation via the phenylpropanoid metabolic and carbohydrate metabolism. In addition, many DEGs were classified into the process for response to light stimulus term, indicating that hematin may also participate in the light stimulus system.

Pathway enrichment analysis was performed to understand the biological functions of the DEGs by identifying significantly enriched signal transduction pathways or metabolic pathways [39]. In the study, a number of altered biological pathways associated with the hematin treatment were identified, and three pathways, including RNA polymerase, pyrimidine metabolism, and purine metabolism, were significantly enriched (*Q*-value < 0.05) (Table 2). Although the majority of the pathway terms were not significantly enriched, the pathway enrichment analysis helps us to further understand the biological functions of the DEGs and the molecular mechanisms that regulate fruit coloring in response to the hematin treatment. These pathway terms included metabolic pathways, biosynthesis of secondary metabolites, isoflavonoid biosynthesis, phenylalanine metabolism, phenylalanine, tyrosine and tryptophan biosynthesis, starch and sucrose metabolism, plant hormone signal transduction, flavone and flavonol biosynthesis, and phenylpropanoid biosynthesis terms (Supplementary Table S2).

### 3.4. Analysis of DEGs Involved in Anthocyanin Biosynthesis

#### 3.4.1. Transcription Factors

Transcription factors (TFs) are proteins that regulate the expression of downstream target genes. Many TFs, such as v-myb avian myeloblastosis viral oncogene homolog (MYB), basic helix-loop-helix (bHLH), WD40-repeats protein (WD40), and MADS-box (MADS), directly regulating the expression of the structural genes in anthocyanin biosynthesis have been identified from many species [8]. Therefore, the enhanced expression of key TFs might regulate the anthocyanin biosynthesis. In our study, some anthocyanin biosynthesis-related TFs were found to be uniquely present in the DEG profiling of the hematin-treated fruit, for example, MYB, bHLH, PIF3, MADs-box, AP2-EREBP, and ABI3/VP1 (Table 3). The anthocyanin biosynthesis genes are regulated primarily by the MBW transcription factor, which is a ternary transcription factor complex [10, 11]. Most of the MYBs involved in regulating anthocyanin biosynthesis are positive regulators of transcription [8]. However, MYBs can act as repressors too, such as strawberry* FaMYB1* and* FaMYB9 *and grapevine* VvMYB4, *which can significantly suppress the biosynthesis of anthocyanins and flavonols [40]. In this study, three unigenes belonging to MYB transcription factors were differentially expressed, and two MYB transcription factors were significantly upregulated. Of these, the unigene “FANhyb_rscf00000146.1.g00007.1” from the* MYB* family of R2R3 MYB transcription factors was significantly upregulated. Moreover, increasing evidence indicates that the expression of the bHLHs promotes anthocyanin accumulation in fruit [41]. In our study, all of the unigenes from the bHLH family were downregulated, among which the expression level of unigene FANhyb_rscf00001292.1.g00003.1 was significantly decreased 6250-fold (Table 3). FANhyb_rscf00002210.1.g00001.1 encodes a predicted MADS-box transcription factor, and it was significantly downregulated. There were three unigenes in the AP2-EREBP family, with only one upregulated and the others downregulated. These results suggest that exogenous hematin is involved in developmental transcriptional regulation of anthocyanin biosynthesis.

#### 3.4.2. DEGs in Anthocyanin Biosynthesis

The anthocyanin biosynthetic pathway has been studied intensively. The anthocyanin biosynthetic pathway via the phenylpropanoid pathway is well known [8]. In this study, the transcriptome data showed a significant increase in the expression of genes in the anthocyanin biosynthesis pathway when exposed to exogenous hematin. The structural genes involved in anthocyanin biosynthesis, including DFR, LAR, and UDP-glycosyltransferase, exhibited significant differential expression in response to the hematin treatment (Table 4). For example, two* DFR* genes and one* LAR* gene were significantly upregulated (the expression increased 18-, 30-, and 3-fold in the hematin-treated fruit, resp.). In addition, all six genes encoding* UDP-glycosyltransferase *were significantly upregulated. Among these, the expression of one gene (Fanhy_icon15742070o.1.g00001.1) was upregulated 18305-fold. The results suggest that exogenous hematin may have an important role in promoting anthocyanin biosynthesis.

#### 3.4.3. DEGs in Hormone Signaling Transduction in Response to Hematin

Auxin has been shown to negatively regulate the expression of the anthocyanin biosynthesis genes [42]. In this study, there were three unigenes involved in the auxin signaling pathway, and all were downregulated, including one gene encoding auxin efflux carrier component 1-like (AUX1-like) and two genes encoding auxin response factors (ARF). These results suggest that hematin promotes anthocyanin accumulation by regulating auxin in the coloration process of strawberries. It has shown that auxin suppresses anthocyanin biosynthesis in the red-fleshed apple callus [43]. Endogenous expression of auxin [44, 45] has been found to impede anthocyanin accumulation in strawberries. In additional, cytokinins are also known to play an important role in anthocyanin biosynthesis. It has shown to enhance anthocyanin accumulation in* Zea mays* and regulate anthocyanin production and composition in suspension cultures of strawberry cells [46]. In this study, two unigenes related to the cytokinin signaling pathway were identified, and they were downregulated as well. One gene was identified as cytokinin dehydrogenase 5-like which catalyzes the irreversible degradation of cytokinins. This result indicates that hematin promotes anthocyanin accumulation by regulating the cytokinin signaling pathway.

Extensive researches have shown that ABA plays an important role in the regulation of anthocyanin biosynthesis in nonclimacteric fruit [47]. In this study, a total of five unigenes related to the ABA signaling pathway were identified, among which two were upregulated and three were downregulated. Two genes, which are involved in the abscisic acid response, were upregulated. These two unigenes encode 9-cis-epoxycarotenoid dioxygenase 1 (NCED1) and ABA overly sensitive 5 (ABO5), respectively. ABA is synthesized from carotenoids via several enzymatic reactions in the plastid. The rate-limiting step in these reactions is catalyzed by 9-cis-epoxycarotenoid dioxygenase 1 (NCED1) [48]. Mutations in the* FaNCED1 *gene result in colorless strawberry fruit, which can become colored through the application of exogenous ABA [12]. Another unigene was identified to encode abscisic acid 8′-hydroxylase 1-like, a key enzyme in the oxidative catabolism of abscisic acid, which was downregulated. Application of exogenous ABA regulates phenylalanine ammonia lyase activity and increases the phenolic and anthocyanin content of strawberry fruit [12, 49]. Our results suggest that hematin promotes ABA biosynthesis and inhibits ABA disintegration in the hematin-treated fruit.

In* Arabidopsis*, JAs can affect anthocyanin accumulation via the interaction of negative regulators with the MBW complex of transcription factors involved in anthocyanin biosynthesis [50]. Preharvest application of JA to “*Fuji*” apples enhances red coloration [51]. JA vapor treatment can also enhance anthocyanins in strawberry fruit [52]. In this study, jasmonate O-methyltransferase-like exhibited significantly differential expression. Jasmonate O-methyltransferase-like is also known to be a key enzyme for jasmonate-regulated plant responses to stimuli [53, 54]. These results suggest that hematin increases the activity of the JA-regulated anthocyanin biosynthesis.

#### 3.4.4. DEGs in the Phytochrome Signaling Pathway in Response to Hematin

Phytochromes that act as photoreceptors play an important role in anthocyanin regulation [15]. Phytochromes are homodimeric chromoproteins, where each holophytochrome is composed of a phytochrome protein (apophytochrome) covalently bound to a linear tetrapyrrole PΦB. HO1 is crucial to this process and acts as a rate-limiting enzyme in the biosynthesis of PΦB [55]. Exogenous hematin can also induce* HO-1* expression in many plants [56, 57]. In this study, we found exogenous hematin significantly increased the expression of* FaHO1* (Table 5). This result implies that hematin can promote PΦB biosynthesis. Recently, it has been reported that hematin could induce the accumulation of far-red phytochrome and* phytochrome A* transcripts in the etiolated leaves of wheat seedling phytochromes [26]. However, our study shows that phytochrome A was not differentially expressed. It is probably because the hematin-treated strawberries were not shaded during the experiment, while the function of phytochrome A is light-dependent. In addition, we found that a downstream gene of phytochrome, PIF3 (FANhyb_rscf00000669.1.g00002.1), was significantly upregulated. PIF3 is thought to be a positive regulator of phytochrome B mediated by light-dependent signal transduction [58]. These results suggest that the phytochrome pathways may be involved in the anthocyanin biosynthesis promoted by hematin.

#### 3.4.5. DEGs in Starch and Sucrose Degradation

Proteomic approaches have revealed that starch degradation contributes to anthocyanin accumulation in tuberous roots of the purple sweet potato variety [59]. Starch and sucrose biosynthesis is important for anthocyanin accumulation in strawberry fruit [60]. In this study, we found the DEGs involved in starch and sucrose synthesis in the hematin-treated fruit were all downregulated (Table 5). For example, the expression of the unigenes encoding soluble starch synthase 3, sucrose transport protein SUC2-like, and a probable sucrose-phosphate synthase 4-like enzyme were decreased 2.3-, 4.6-, 3.2-, and 2-fold, respectively. This suggests that exogenous hematin regulates the biosynthesis of starch and sucrose and hence affects fruit coloring.

#### 3.4.6. DEGs in the Calcium Pathway in Response to Hematin

Calcium can also increase the transcription levels of key structural genes* F3H*,* DFR*,* ANT,* and* UFGT *in the white stage of strawberries [61]. In this study, we found that three DEGs in calcium biosynthesis and transport were downregulated in the hematin-treated fruit (Table 5). Only the unigene encoding predicted cation/calcium exchanger 5-like was upregulated. The unigenes encoding a calpain-type cysteine protease and a predicted calcium-binding protein PBP1-like were downregulated, respectively. Exogenous application of calcium can promote apple coloring [62] in addition to variation in anthocyanin content [61]. Our results indicate that the biosynthesis and transport of calcium are involved in the development of coloring in hematin-treated strawberries.

### 3.5. Validation of Selected DEGs by qRT-PCR

**Figure 4:**
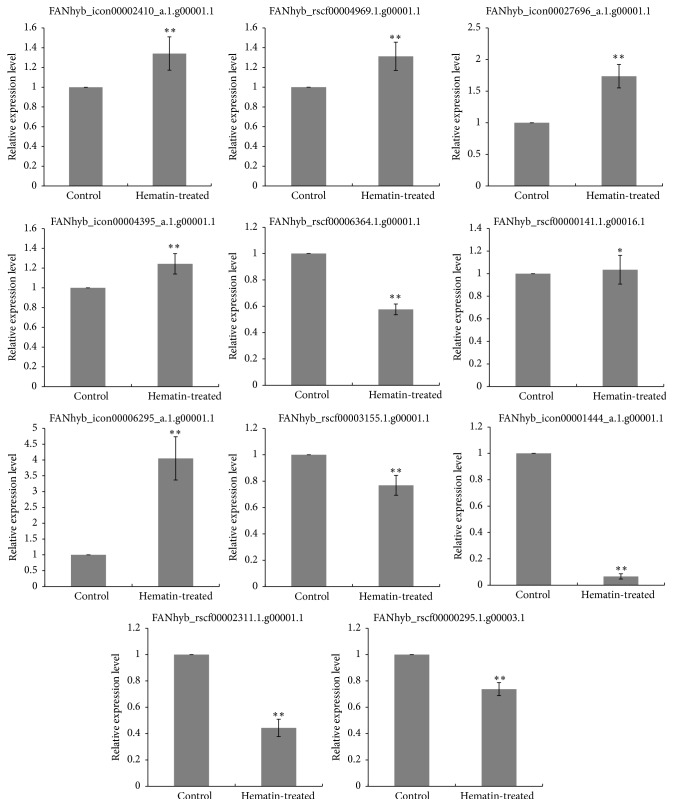
qRT-PCR validation of the RNA-Seq based gene expression. The values indicate means of three biological replicates ± SD. Star indicates that the expression level is significantly different between the hematin-treated and the control group (^*∗*^ *p* < 0.05, ^*∗∗*^ *p* < 0.01).

To validate the expression of the DEGs obtained from RNA-Seq, 11 DEGs were selected for qRT-PCR, including structural genes (*DFR* and* UDFGs*), transcription factor genes (*MYB* and* bHLH*), and phytochrome chromophore-related gene (*HO-1*). The primers used for qRT-PCR are listed in Supplementary Table S1. The qRT-PCR results were consistent with the RNA-Seq data (Figure 4), except for FANhyb_rscf00000141.1.g00016.1, which had a higher log_2_ ratio (hematin-treated/control) in the transcriptome data than in qRT-PCR. These results indicate the RNA-Seq data from the strawberry transcriptome is reproducible and accurate.

## 4. Conclusions

**Figure 5:**
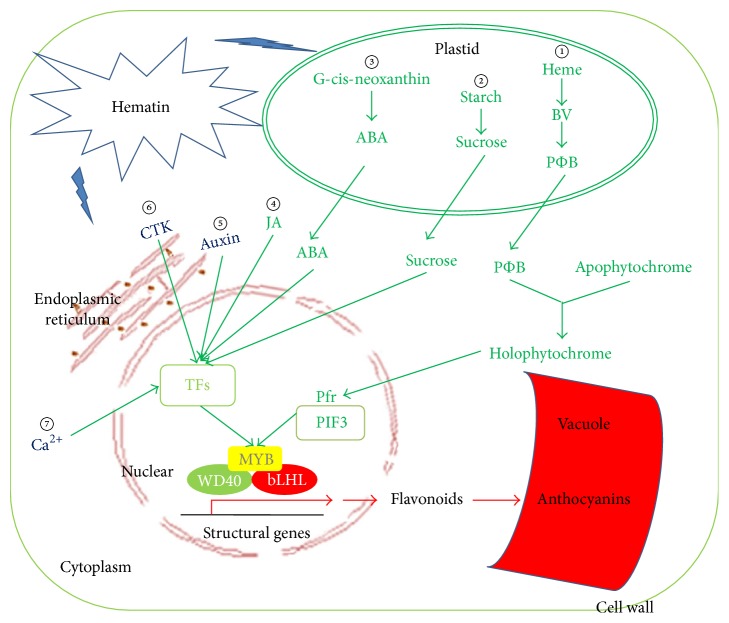
Genes and biological pathways that were involved in regulation of anthocyanin accumulation promoted by hematin. Outermost box represents the cell wall. Bilayer oval shape represents the plastid. The red-brown ring and network represent the nuclear and endoplasmic reticulum, respectively. The red crescent represents the vacuole. The box in the nucleus represents transcription factors (TFs). Yellow MYB, green WD40, and red bHLH represent a ternary transcription factor complex which transcribes anthocyanin biosynthesis genes. The circled numbers represent biological pathways involved in the regulation of anthocyanin accumulation promoted by hematin. ① represents the phytochrome regulation pathway. ② represents the starch and sucrose pathway. ③, ④, ⑤, and ⑥ represent phytohormones ABA, JA, Auxin, and CTK regulation pathways, respectively. ⑦ represents the Ca^2+^ regulation pathway. Pfr represents the far-red phytochrome. PIF3 represents phytochrome-interacting factor. Each anthocyanin biosynthesis regulatory pathway related gene is in Table 5.

In this study, the anthocyanin content in the strawberry fruit was elevated by the application of exogenous hematin. This is the first report that hematin could increase the anthocyanin production in fruit. Furthermore, we explored the effects of the exogenous hematin on metabolic pathways using genome-wide transcriptome analysis. The results indicate that the expression levels of many genes involved in anthocyanin biosynthesis were significantly altered with the hematin treatment. This suggests that the physiological process of fruit color development is regulated through complex interactions among anthocyanin biosynthesis pathways, plant hormone signal transduction pathways, phytochrome signal transduction pathways, starch and sugar metabolic pathways, calcium pathways, and transcription factors (Figure 5). This study adds to the in-depth understanding of the fruit coloration process in strawberry.

## Supplementary Material

Figure S1. The anthocyanin content in strawberry fruit was measured after treatment with different concentrations of hematins (0, 1, 10 and 100μM). Vertical bars represent standard errors; Values with different letter are significantly different at p<0.01. Figure S2. Saturation analysis of control 1, control 2, hematin 1, and hematin 2 mRNA libraries. (TIFF); Figure S3. Randomness assessments of control 1, control 2, hematin 1, and hematin 2 mRNA libraries. (TIFF); Table S1. Eleven pairs of gene specific primers of the DEGs in strawberry fruit. Table S2. Gene classification based on gene ontology and pathway terms for DEGs treated by hematin.

## Figures and Tables

**Table 1:** Overview of the sequencing and assembly.

	*Biological replicate 1*		*Biological replicate 2*	
Sample	Control	Hematin-treated	Control	Hematin-treated
Clean reads	17808924	26809908	19235452	26228671
Genome map rate (%)	96.58	96.52	96.65	96.74
Gene map rate (%)	86.46	86.27	87.04	85.63
Perfect match (%)	4677273 (24.32%)	6510668 (24.28%)	4414050 (24.79%)	6149508 (23.45%)
Unique match (%)	15075148 (84.65%)	22628939 (84.41%)	16409557 (85.31%)	21986962 (83.83%)
Expressed genes	27976	28713	28999	28211
Mismatch (%)	11321162 (63.57%)	17101590 (63.79%)	12456871 (64.76%)	16697280 (63.66%)
Total unmapped reads	645135 (3.35%)	932213 (3.48%)	608914 (3.42%)	856028 (3.26%)

**Table 2:** Pathway enrichment analysis of differentially expressed genes.

Pathway	DEGs with pathway annotation (451)	All genes with pathway annotation (15877)	Corrected *p* value	*Q*-value	Pathway ID
RNA polymerase	16	183	6.88*E* − 5	0.0068107	ko03020
Pyrimidine metabolism	20	317	0.0007651746	0.0320	ko00240
Purine metabolism	21	347	0.0009710371	0.032044224	ko00230

**Table 3:** DEGs acting as transcription factors in response to exogenous hematin.

Annotation transcription factors	Gene ID	Real fold-change values	Upregulation/downregulation	FDR
PREDICTED: AP2-like ethylene-responsive transcription factor AIL1-like	FANhyb_icon00006295_a.1.g00001.1	9.38	Up	1.20*E* − 05
PREDICTED: AP2-like ethylene-responsive transcription factor AIL1-like	FANhyb_rscf00003155.1.g00001.1	4	Down	4.80*E* − 16
PREDICTED: AP2-like ethylene-responsive transcription factor AIL5-like	FANhyb_rscf00000029.1.g00010.1	4	Down	3.20*E* − 08
MYBdomain protein 66	FANhyb_rscf00000146.1.g00007.1	6.54	Up	7.80*E* − 07
MYB-related protein 3R-1-like, partial	FANhyb_rscf00000141.1.g00016.1	2.82	Up	8.30*E* − 04
PREDICTED: transcription factor GAMYB-like	FANhyb_rscf00000649.1.g00006.1	3.03	Down	1.50*E* − 13
PREDICTED: probable WRKY transcription factor 53-like	FANhyb_rscf00001973.1.g00002.1	2.17	Down	2.00*E* − 08
Transcription factor bHLH80-like	FANhyb_rscf00001292.1.g00003.1	6250	Down	1.50*E* − 06
Transcription factor bHLH70-like	FANhyb_rscf00000295.1.g00003.1	5.26	Down	6.60*E* − 12
Transcription factor ICE1-like	FANhyb_rscf00000170.1.g00006.1	2.44	Down	4.50*E* − 18
C2H2-like zinc finger protein	FANhyb_rscf00001143.1.g00002.1	2.82	Up	5.90*E* − 04
Zinc finger protein MAGPIE-like	FANhyb_rscf00003024.1.g00001.1	2.04	Up	1.50*E* − 04
PREDICTED: axial regulator YABBY 5-like	FANhyb_rscf00001667.1.g00001.1	4.17	Down	7.30*E* − 05
PREDICTED: axial regulator YABBY 1-like	FANhyb_rscf00006899.1.g00001.1	3.57	Down	6.20*E* − 06
PREDICTED: dof zinc finger protein DOF3.7-like isoform 1	FANhyb_rscf00006856.1.g00001.1	2.63	Down	2.80*E* − 04
ZF-HD homeobox protein	FANhyb_rscf00000044.1.g00022.1	3.85	Down	4.20*E* − 08
ZF-HD homeobox protein At4g24660-like	FANhyb_rscf00005241.1.g00001.1	3.57	Down	4.70*E* − 09
PREDICTED: B3 domain-containing protein REM14-like	FANhyb_rscf00001009.1.g00002.1	2.43	Up	5.30*E* − 04
PREDICTED: B3 domain-containing transcription factor ABI3-like	FANhyb_rscf00001271.1.g00002.1	4.55	Down	1.30*E* − 28
Growth-regulating factor 6	FANhyb_rscf00000024.1.g00029.1	3.45	Down	3.30*E* − 34
Growth-regulating factor 5	FANhyb_icon00009704_a.1.g00001.1	2.7	Down	1.90*E* − 06
GATA type zinc finger transcription factor family protein	FANhyb_rscf00000393.1.g00013.1	11.11	Down	1.10*E* − 05
PREDICTED: zinc finger CCCH domain-containing protein 2-like	FANhyb_icon00006770_a.1.g00001.1	3.13	Down	6.80*E* − 49
PREDICTED: E2F transcription factor-like E2FE-like	FANhyb_icon00002702_a.1.g00001.1	2.78	Down	2.40*E* − 04
PREDICTED: uncharacterized protein	FANhyb_rscf00001066.1.g00001.1	2.63	Down	4.90*E* − 06
PREDICTED: nuclear transcription factor Y subunit A-3-like isoform 1	FANhyb_rscf00000008.1.g00035.1	3.85	Down	5.00*E* − 20
PREDICTED: homeobox-leucine zipper protein ATHB-8-like	FANhyb_rscf00000015.1.g00005.1	2.63	Down	1.00*E* − 06
PREDICTED: MADS-box transcription factor 18-like	FANhyb_rscf00000323.1.g00016.1	2.43	Down	1.80*E* − 06
PREDICTED: zinc finger protein 132-like isoform 1	FANhyb_rscf00007015.1.g00001.1	2.43	Down	1.80*E* − 08
PREDICTED: transcription factor PIF3-like	FANhyb_rscf00000669.1.g00002.1	3.46	Up	1.40*E* − 07

**Table 4:** Regulation of the DEGs in anthocyanin biosynthesis in response to exogenous hematin.

Genes in anthocyanin biosynthesis	Gene ID	Real fold-change values	Regulation level	FDR
Dihydroflavonol-4-reductase	FANhyb_icon00002410_a.1.g00001.1	30.91	Up	9.10*E* − 06
Flavonol synthase	FANhyb_icon00015354_a.1.g00001.1	3.45	down	1.80*E* − 05
Isoflavone reductase homolog	FANhyb_icon20341135_s.1.g00001.1	3.34	Up	7.50*E* − 14
UDP-glycosyltransferase	FANhyb_rscf00004969.1.g00001.1	3.07	Up	6.40*E* − 08
UDP-glycosyltransferase activity	FANhyb_icon15742070_o.1.g00001.1	18 305.63	Up	5.10*E* − 09
UDP-glycosyltransferase 73C1	FANhyb_icon00027696_a.1.g00001.1	2.98	Up	5.60*E* − 04
UDP-glycosyltransferase activity	FANhyb_icon00008064_a.1.g00001.1	2.49	Up	3.10*E* − 04
PREDICTED: UDP-glucose flavonoid 3-O-glucosyltransferase 7-like	FANhyb_rscf00005563.1.g00001.1	2.12	Up	1.10*E* − 19
PREDICTED: UDP-glycosyltransferase 76F1-like	FANhyb_rscf00002177.1.g00003.1	2	Up	7.20*E* − 29

**Table 5:** Other DEGs identified in anthocyanin biosynthesis-related pathways.

Anthocyanin biosynthesis-related pathways	Annotation genes	Gene ID	Real fold-change values	Upregulation/downregulation	FDR
Calcium ion binding	Cation/calcium exchanger 5-like	FANhyb_rscf00001906.1.g00001.1	2.03	Up	6.4*E* − 06
	Calpain-type cysteine protease family	FANhyb_icon00011755_a.1.g00001.1	3.45	Down	1.8*E* − 09
	Calcium-binding protein PBP1-like	FANhyb_rscf00000750.1.g00008.1	3.23	Down	9.7*E* − 04
	Calpain-type cysteine protease family	FANhyb_icon00023658_a.1.g00001.1	2.33	Down	6.0*E* − 08

Cytokinin	Cytokinin dehydrogenase 5-like	FANhyb_iscf00325393_1_s.1.g00001.1	6.67	Down	6.2*E* − 06
	Cytochrome P450 714A1	FANhyb_rscf00000592.1.g00003.1	2.17	Down	9.8*E* − 10

Jasmonate	Jasmonate O-methyltransferase-like	FANhyb_rscf00000004.1.g00013.1	2.01	Up	7.0*E* − 04

Abscisic acid	Protein ABSCISIC ACID-INSENSITIVE 5-like	FANhyb_rscf00002164.1.g00001.1	4	Down	1.6*E* − 18
	ABA overly sensitive 5	FANhyb_icon00051144_a.1.g00001.1	2.91	Up	2.4*E* − 05
	9-cis-epoxycarotenoid dioxygenase NCED1	FANhyb_icon18399909_o.1.g00001.1	4.41	Up	4.3*E* − 06
	Abscisic acid 8′-hydroxylase 1-like	FANhyb_icon00000938_a.1.g00001.1	2.56	Down	1.4*E* − 06
	Abscisic acid receptor PYR1-like	FANhyb_icon00020426_a.1.g00001.1	2.08	Down	3.0*E* − 06

Auxin	Auxin efflux carrier component 1-like	FANhyb_rscf00001008.1.g00003.1	3.84	Down	2.3*E* − 06
	Auxin response factor 8	FANhyb_rscf00001306.1.g00002.1	2.63	Down	6.1*E* − 17
	Auxin response factor 17-like	FANhyb_rscf00006074.1.g00001.1	2.17	Down	1.1*E* − 36

Starch and sucrose	Glycosyltransferase, family 35	FANhyb_rscf00000034.1.g00008.1	3.84	Down	1.1*E* − 05
	Soluble starch synthase 3	FANhyb_rscf00000045.1.g00004.1	2.32	Down	3.8*E* − 12
	Sucrose transport protein SUC2-like	FANhyb_icon00036727_a.1.g00001.1	4.55	Down	9.8*E* − 04
	Sucrose transport protein SUC2-like	FANhyb_rscf00000755.1.g00002.1	3.23	Down	6.6*E* − 10
	Probable sucrose-phosphate synthase 4-like	FANhyb_rscf00001350.1.g00002.1	2.13	Down	1.1*E* − 14

Phytochrome	PREDICTED: transcription factor PIF3-like	FANhyb_rscf00000669.1.g00002.1	3.46	up	1.4*E* − 07
	Phytochrome E-like	FANhyb_rscf00000436.1.g00004.1	4	Down	3.3*E* − 05
	Heme oxygenase 1	FANhyb_icon00004395_a.1.g00001.1	2.58	2.7*E* − 07	2.7*E* − 07
