# Supplementary figures and images for: Comparative Transcriptome Analysis Reveals Effects of Exogenous Hematin on Anthocyanin Biosynthesis during Strawberry Fruit Ripening
Source: Int J Genomics. 2016 Dec 15;2016:6762731. doi: 10.1155/2016/6762731 (PMC5198259; doi:10.1155/2016/6762731)

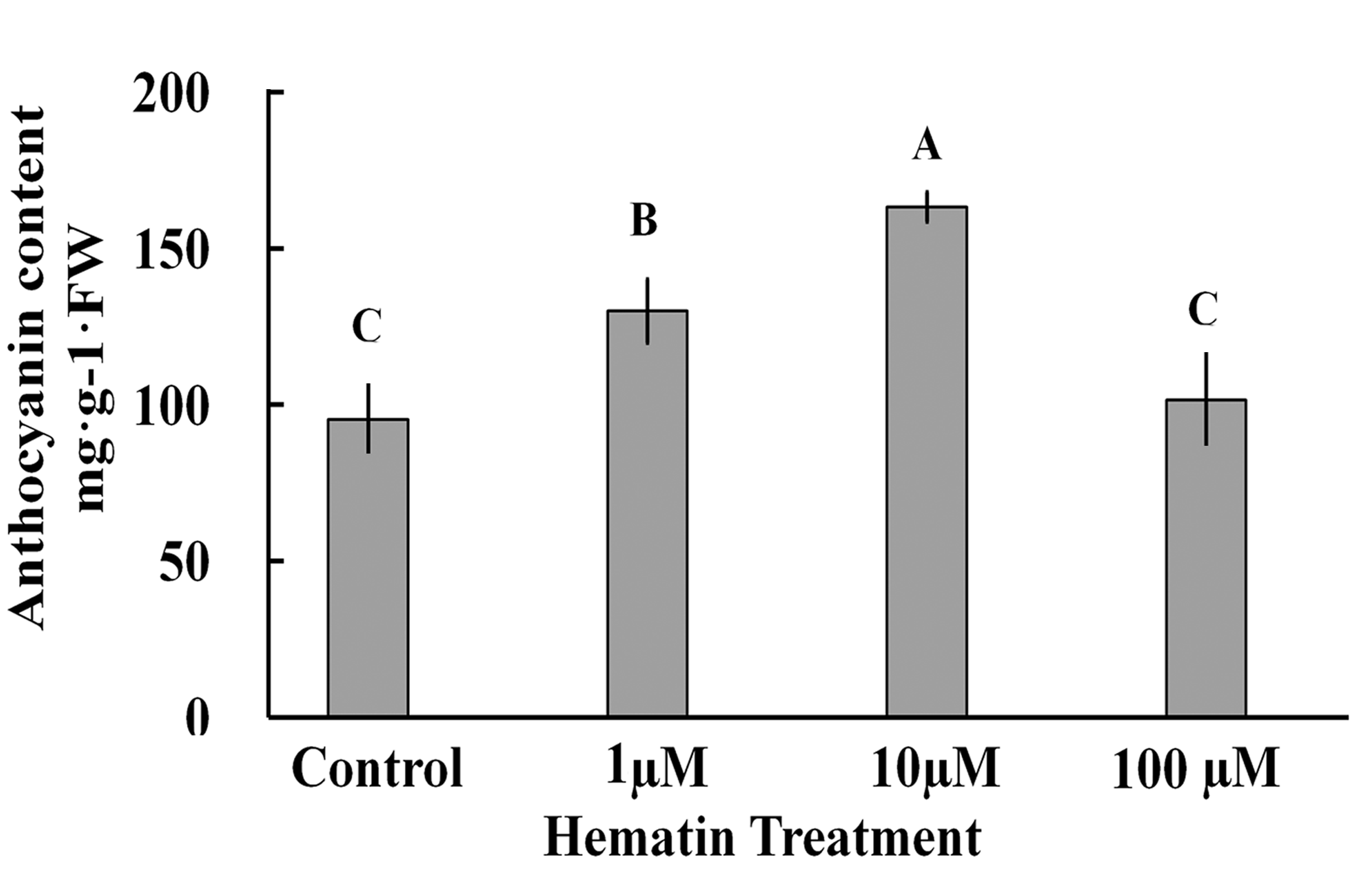

Supplement: Supplementary file 1 — Figure S1. The anthocyanin content in strawberry fruit was measured after treatment with different concentrations of hematins (0, 1, 10 and 100μM). Vertical bars represent standard errors; Values with different letter are significantly different at p<0.01. Figure S2. Saturation analysis of control 1, control 2, hematin 1, and hematin 2 mRNA libraries. (TIFF); Figure S3. Randomness assessments of control 1, control 2, hematin 1, and hematin 2 mRNA libraries. (TIFF); Table S1. Eleven pairs of gene specific primers of the DEGs in strawberry fruit. Table S2. Gene classification based on gene ontology and pathway terms for DEGs treated by hematin. [file 6762731.f1.zip › supplementary materials/Figure S1.png]

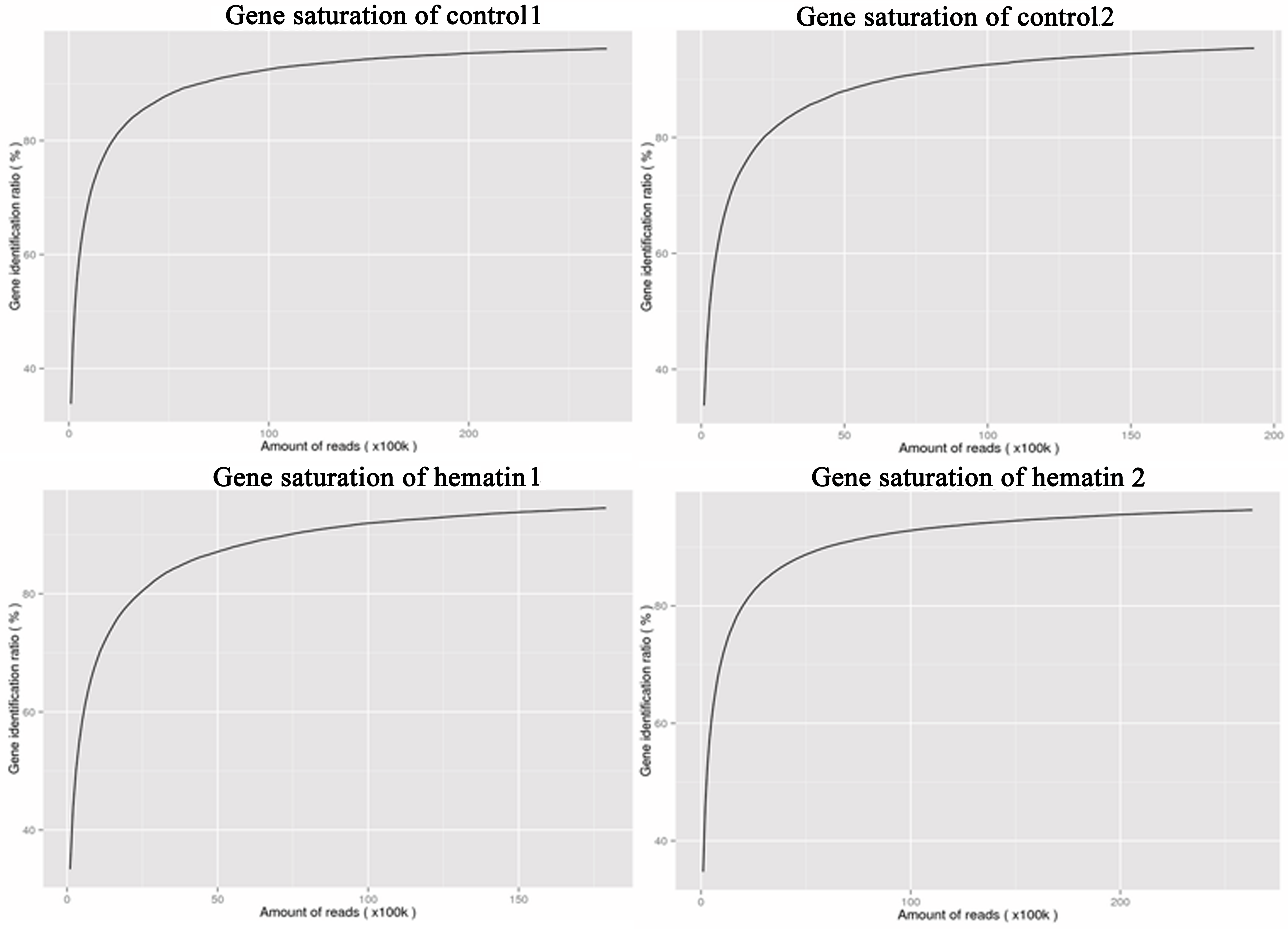

Supplement: Supplementary file 1 — Figure S1. The anthocyanin content in strawberry fruit was measured after treatment with different concentrations of hematins (0, 1, 10 and 100μM). Vertical bars represent standard errors; Values with different letter are significantly different at p<0.01. Figure S2. Saturation analysis of control 1, control 2, hematin 1, and hematin 2 mRNA libraries. (TIFF); Figure S3. Randomness assessments of control 1, control 2, hematin 1, and hematin 2 mRNA libraries. (TIFF); Table S1. Eleven pairs of gene specific primers of the DEGs in strawberry fruit. Table S2. Gene classification based on gene ontology and pathway terms for DEGs treated by hematin. [file 6762731.f1.zip › supplementary materials/Figure S2.png]

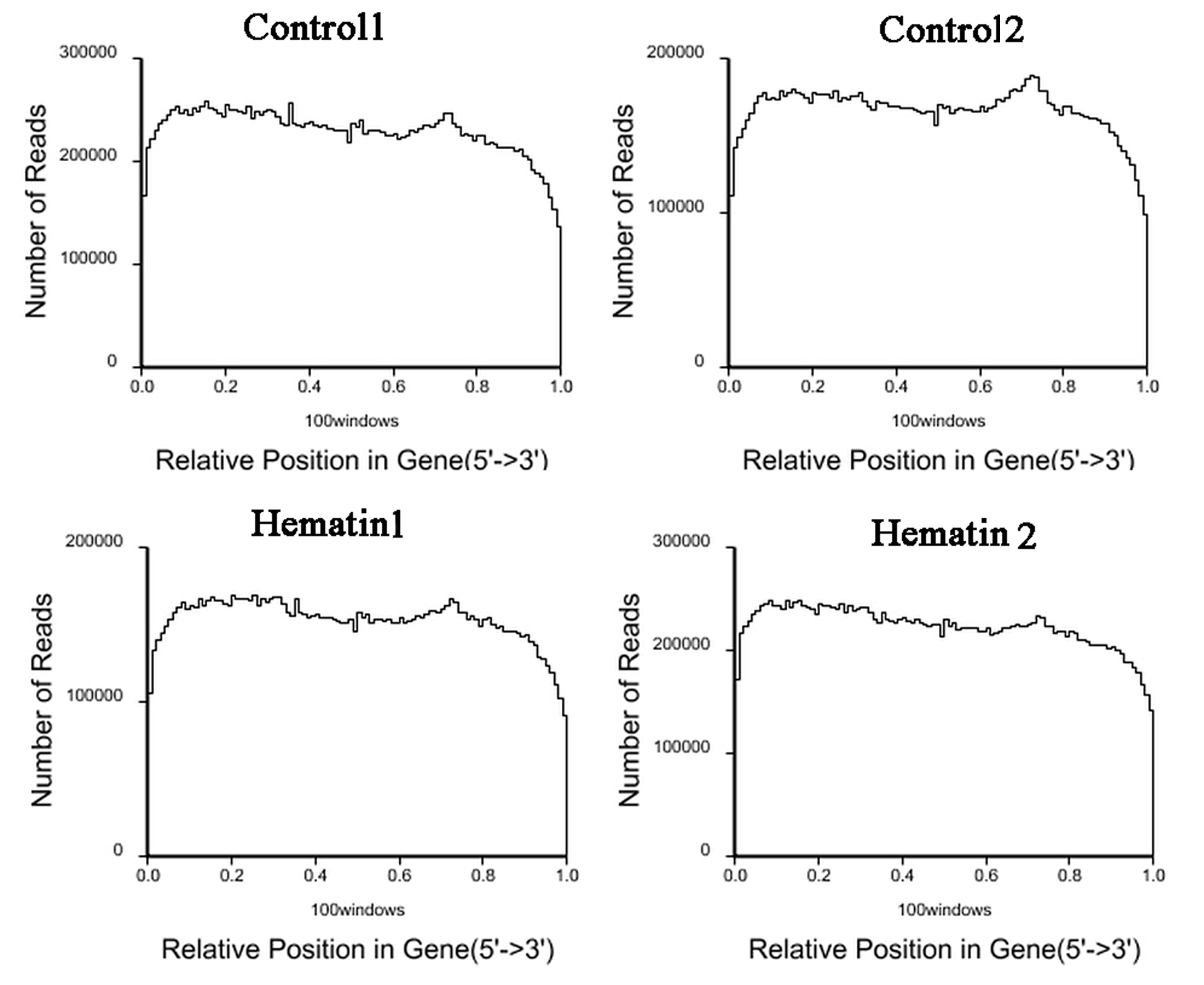

Supplement: Supplementary file 1 — Figure S1. The anthocyanin content in strawberry fruit was measured after treatment with different concentrations of hematins (0, 1, 10 and 100μM). Vertical bars represent standard errors; Values with different letter are significantly different at p<0.01. Figure S2. Saturation analysis of control 1, control 2, hematin 1, and hematin 2 mRNA libraries. (TIFF); Figure S3. Randomness assessments of control 1, control 2, hematin 1, and hematin 2 mRNA libraries. (TIFF); Table S1. Eleven pairs of gene specific primers of the DEGs in strawberry fruit. Table S2. Gene classification based on gene ontology and pathway terms for DEGs treated by hematin. [file 6762731.f1.zip › supplementary materials/Figure S3.tif]
